# β-cyclodextrin chitosan-based hydrogels with tunable pH-responsive properties for controlled release of acyclovir: design, characterization, safety, and pharmacokinetic evaluation

**DOI:** 10.1080/10717544.2021.1921074

**Published:** 2021-06-11

**Authors:** Nadia Shamshad Malik, Mahmood Ahmad, Mohammed S. Alqahtani, Arshad Mahmood, Kashif Barkat, Muhammad Tariq Khan, Ume Ruqia Tulain, Ayesha Rashid

**Affiliations:** a Faculty of Pharmacy, Capital University of Science and Technology, Islamabad, Pakistan; b Faculty of Pharmacy, University of Central Punjab, Lahore, Pakistan; c Department of Pharmaceutics, Nanobiotechnology Unit, College of Pharmacy, King Saud University, Riyadh, Saudi Arabia; d College of Pharmacy, Al Ain University, Abu Dhabi, UAE; e Faculty of Pharmacy, University of Lahore, Lahore, Pakistan; f Faculty of Pharmacy, University of Sargodha, Sargodha, Pakistan; g Department of Pharmacy, The Women University, Multan, Pakistan

**Keywords:** Hydrogel, acyclovir, β-cyclodextrin, chitosan, methacrylic acid, acute oral toxicity

## Abstract

In this work, series of pH-responsive hydrogels (FMA1–FMA9) were synthesized, characterized, and evaluated as potential carrier for oral delivery of an antiviral drug, acyclovir (ACV). Different proportions of β-cyclodextrin (β-CD), chitosan (CS), methacrylic acid (MAA) and N′ N′-methylenebis-acrylamide (MBA) were used to fabricate hydrogels *via* free radical polymerization technique. Fourier transform infrared spectroscopy confirmed fabrication of new polymeric network, with successful incorporation of ACV. Scanning electron microscopy (SEM) indicated presence of slightly porous structure. Thermal analysis indicated enhanced thermal stability of polymeric network. Swelling studies were carried out at 37 °C in simulated gastric and intestinal fluids. The drug release data was found best fit to zero-order kinetics. The preliminary investigation of developed hydrogels showed a pH-dependent swelling behavior and drug release pattern. Acute oral toxicity study indicated no significant changes in behavioral, clinical, or histopathological parameters of Wistar rats. Pharmacokinetic study indicated that developed hydrogels caused a significant increase in oral bioavailability of ACV in rabbit plasma as compared to oral suspension when both were administered at a single oral dose of 20 mg kg^−1^ bodyweight. Hence, developed hydrogel formulation could be used as potential candidate for controlled drug delivery of an antiviral drug acyclovir.

## Introduction

Hydrogels are three-dimensional networks of hydrophilic polymers, which can absorb water or physiological medium and swell up to thousands of time of their initial dry weights (Hoffman, 2012).

They are formed by natural, synthetic, or combination of both polymers *via* numerous ways of crosslinking (Caló and Khutoryanskiy, 2015). These gels resemble like biological tissues due to soft and rubbery nature in the swollen state (Peppas and Hoffman, 2020). The protection of drug from hostile environment of the body is also a significant advantage of hydrogel. Hydrogels are stimuli sensitive networks and change the gel structure in response to various stimuli, such as pH, temperature, light, electric/magnetic field, as well as specific molecular recognition. There is a considerable interest in fabricating hydrogel drug carriers using natural polymers due to their nontoxicity, biodegradability, and biocompatibility (Ullah et al., 2015). Unfortunately, major drawbacks associated with these natural polymer-based hydrogels include their weak mechanical strength and the burst release of drugs. However, different natural or synthetic polymers can be integrated together to develop polymeric carriers with some special characteristics, such as moderate swelling capacity and stimuli-responsive features, which are useful for drug release systems (Hu et al., 2017).

Chitosan (CS), a cationic biopolymer, consists of β-(1-4)-2-acetamido-2-deoxy-D-glucose unit, which is the main alkaline deacetylation product of chitin. CS has an advantage over other polysaccharides due to its nontoxicity, biocompatibility, and biodegradability (Dinu et al., 2016). The presence of hydroxyl and amine groups which are good cross-linkable sites for various cross-linking agents to prepare inter-penetrating network (IPN) structures (Chen et al., 2016). Thus, in order to broaden its exploitation in biomedical field, CS can be cross linked with different synthetic polymers or monomers to prepare drug carriers with improved mechanical strength, thermal stability, desirable swelling, and drug release profile in stable hydrogel network (Qu and Luo, 2020).

Cyclodextrins (CDs) are series of oligosaccharides produced by *Bacillus macerans* during the enzymatic degradation process of starch and related compounds. Among various CDs, β-cyclodextrin (β-CD), a natural molecule derived from starch, is a torus-shaped cyclic oligosaccharide (Machín et al., 2012). As a result of electron-rich glycosidic oxygen atoms, the interior of toroid or central cavity is hydrophobic. Consequently, hydrophobic molecules or groups can be included into cavities of β-CD in the presence of water (Jiang et al., 2010). Moreover, β-CD is known to enhance the solubility of incorporated drug and to act as permeation enhancers for different macromolecular drugs (Pinho et al., 2014).

Acyclovir (ACV), a synthetic nucleoside analog derived from guanosine, is a drug of choice and common in clinical use for the treatment of herpes virus infection (Perret et al., 2013). However, after oral administration, absorption in the gastrointestinal (GIT) tract is slow and incomplete and its oral bioavailability is quiet less, i.e. 10–30% (Malik et al., 2017a,b). Moreover, neither oral nor the parenteral administration of the currently marketed formulations of ACV is able to achieve desired therapeutic concentrations at target sites. In general, around 80% of the administered dose is not absorbed. Therefore, current marketed formulations require the administration of higher doses, up to 1.2 g/d to achieve desired therapeutic effects. As a consequence, the presence of systemic toxicity and adverse reactions is frequent with its administration (Lembo et al., 2013). Thus, the formulation should be designed in a manner such that the daily dose and dose-related side effects of ACV should be minimized.

In recent years, many technological approaches have been proposed for decreasing the drawbacks associated with conventional dosage forms of ACV and to improve its efficacy. One strategy is to incorporate ACV in polymeric drug delivery system, such as hydrogels. This work, therefore, focuses on the potential use of β-CD and CS as polymeric carriers in the form of hydrogels for controlled drug delivery of ACV.

Several hydrogels were prepared using various concentration of β-CD, CS, MAA, and KPS by free radical polymerization technique. The hydrogels have been characterized *via* FTIR, DSC, TGA, PXRD, and SEM. The effect of concentration of reactants on swelling behavior, loading efficiency, and *in vitro* drug release were also examined. Finally, acute oral toxicity study was performed to confirm safety of developed hydrogel.

## Materials and method

### Materials

ACV was obtained from Brooks Pharmaceuticals (Pvt) Ltd. Karachi, Pakistan as donation. β-CD, CS (medium molecular weight, MW 190–310 kDa, degree of deacetylation 75–85%, viscosity 200–800 cP) and N′, N′-methylenebis-acrylamide (MBA) were purchased from Sigma-Aldrich (St. Louis, MO). Methacrylic acid (MAA) was obtained from Sigma-Aldrich (UK). The analytical grade initiator potassium persulfate (KPS) was obtained from Fluka (Germany). Deionized distilled water was freshly prepared in research laboratory at the Islamia University of Bahawalpur, Pakistan.

### Synthesis of β-CD/CS-co-poly(MAA) hydrogels

A series of hydrogels (FMA1–FMA9) were prepared with different content of CS, β-CD, N′ N′-methylene bis-acrylamide (MBA), and KPS through free radical polymerization technique. The feed ratio used to generate series of samples, i.e. FMA1–FMA9 was selected after number of trial and error method. First of all, a measured quantity of pure polymer, CS was dissolved in 20 mL of 1% acetic acid solution and stirred for 30 min at 60 °C. Then measured quantity of polymer β-CD was dissolved in 20 mL of distilled water and added drop wise to above prepared CS solution. An initiator, i.e. KPS was added to above prepared polymer solution with continuous stirring to generate free radicals. The dissolved oxygen of reaction mixture was removed by purging nitrogen gas for half an hour from this reaction mixture. On the other hand, specific amount of monomer (MAA) and crosslinking agent (MBA) were dissolved together. Then reaction mixture of MAA and MBA was added drop wise to above prepared solution of polymers with continuous stirring at 40 °C. Final volume was adjusted with distilled water to 100 mL. After this, the mixture was stirred at 1200 rpm at 55 °C for 8 h until clear, homogenous solution was formed. The resulting solution was transferred to dried glass test tubes and positioned in water bath at 65 °C for 12 h. After 12 h, test tubes were placed at room temperature for 2 h for cooling. Prepared hydrogels were cut into uniform size, i.e. 8 mm after removing from test tubes and then washed with ethanol-water mixture (50:50) to eliminate unreacted species. The removal of unreacted components in the formulation was verified by measuring the absorbance of washing solution in the range of 200–800 nm. Once the absorbance was below 0.001 in the whole range, the disks were further dried in vacuum oven at 40 °C for 1 week (Malik et al., 2017a,b). Chemical compositions of developed hydrogel have been shown in Table 1.

**Table 1.** β-CD/CS-co-poly(MA) hydrogels using different concentration of reactants.

Sample code	Polymer		Monomer	Initiator	Cross linker
	g/100 mL		g/100 mL	g/100 mL	g/100 mL
	β-CD	CS	MAA	KPS	MBA
FMA1	1.5	9	40	0.6	0.5
FMA2	1.5	6	30	0.6	0.5
FMA3	1.5	9	20	0.6	0.5
FMA4	6	9	30	0.6	0.5
FMA5	3	9	30	1.2	0.5
FMA6	1.5	12	30	0.6	0.5
FMA7	1.5	9	30	0.6	0.5
FMA8	1.5	9	30	0.6	0.5
FMA9	1.5	9	30	2.4	0.5

### Drug loading

Drug loading was carried out by immersing dried hydrogels in ACV 1% solution at 25 °C. The drug solution of specific concentration was prepared using 0.2 M phosphate buffer solution, maintained at pH 7.4. Hydrogels were kept submerged in ACV solution until achievement of constant weight. Then loaded hydrogels were removed from drug solution, flushed with distilled water carefully to eliminate any residual mass of drug on the exterior surface of prepared hydrogels. They were further subjected to drying in vacuum oven at 40 °C (Anwar et al., 2020).

### Characterization

#### Fourier transform infrared spectroscopy (FTIR)

For recording FTIR spectra of the reactants and ACV-loaded hydrogels, crushing of samples was done using KBr at a pressure of 600 kg/cm^2^ to obtain desired pallets. A range between 40,000 cm^−1^ and 600 cm^−1^ was selected for spectral scans by employing Bruker FTIR (Tensor 27 series, Bruker Corporation, Germany) instrument, using attenuated total reflectance (ATR) technology accompanying software OPUS data collection (Hajebi et al., 2019a,b).

#### Scanning electron microscopy (SEM)

Structural morphology of hydrogels was investigated using SEM images. Powdered samples were sputtered with gold and placed on aluminum stub. JEOL analytical scanning electron microscope (JSM-6490A, Tokyo, Japan) was used to conduct scanning. About 15 nm, thickness was kept for gold layer, done by gold sputtering (Li et al., 2019).

#### Thermal analysis

Thermal analysis of polymer, monomer, and hydrogel sample was done on TA instrument Q2000 Series, Thermal Analysis system (TA Instrument, West Sussex, UK). For conducting thermal analysis, samples were heated at the rate of 10 °C/min with a flow rate of 20 mL/min up to 500 °C in a nitrogen atmosphere (Bozoğlan et al., 2020).

#### Powder X-ray diffraction (PXRD) analysis

Powder X-ray diffraction (PXRD) was used to investigate nature of the synthesized hydrogels which could be either amorphous or crystalline. Samples were investigated using X-ray diffractometer (X-Pert, PAN analytical, Almelo, Netherlands). The angle of diffraction was varied from 10° to 50° (Algharib et al., 2020).

#### Swelling studies

For investigating swelling dynamics of developed hydrogel formulation, simulated gastric fluid (SGF) and simulated intestinal fluid (SIF) were used as swelling media. Initially, the developed hydrogel formulations were soaked in simulated media maintained at 37 °C. After specified time period, hydrogels samples have been removed and blotted off cautiously to get rid of any liquid droplets adhered on the surface. Hydrogels in swollen state were weighed at predetermined time interval. They were then subjected to drying until achievement of constant weight. The swelling index was calculated as mentioned in Equation (1).

(1) $$\mathrm{Swelling index}(Q)=\frac{Ms}{Md}\;$$

where *Ms* indicates mass of swollen hydrogels at predetermined time interval and *Md* represents the weight of dried hydrogels (Khalid et al., 2018).

#### Determination of drug-loading efficiency (DLE)

Estimation of drug-loading efficiency (DLE) was carried out by crushing drug-loaded hydrogels of known weights carefully in mortar and pestle. They were then soaked in 100 mL of 0.2 M phosphate buffer solution having pH 7.4 for 24 h. After that, sonication was carried out for 20 min to carry extraction of ACV. Further, removal of polymeric debris was done by centrifugation at 300 rpm. Fresh solvent was used to extract polymeric debris for any adhered drug. Analysis of clear supernatant solution was done for ACV by UV–visible Spectrophotometer at *λ*_max_ value of 256 nm (Hajebi et al., 2019 a,b). Estimation of DLE of the developed hydrogels has been done by using following formula as mentioned in Equation (2).

(2) $$\text{Drug loading efficiency}(\%)=\;\frac{\text{Amount of actual drug in the hydrogel}}{\text{Amount of drug added in the hydrogels}}*100$$

#### *In-vitro* drug release studies and drug release kinetics

*In-vitro release* study of ACV from different formulations of hydrogels was designed in simulated GIT conditions to investigate the drug release behavior in different parts of GIT. For this purpose, SGF (0.1 M HCl, pH 1.2), followed by the SIF (0.2 M potassium dihydrogen phosphate, pH 7.4) were used. The experiment was conducted using a USP dissolution apparatus II (Curio; DL-0609) coupled with six baskets. The speed of stirring was kept at 50 rpm. Sample was weighed and added in 900 mL of media, which was kept at 37 °C. The ACV concentration was determined spectrophotometrically using UV–visible Spectrophotometer at *λ*_max_ value of 256 nm. Release data for various developed hydrogels was evaluated by computing various kinetic models, such as zero-order, first-order, Higuchi, and Korsmeyer–Peppas models (Feki et al., 2020).

### *In-vivo* studies

#### Acute oral toxicity study

##### Animals

Ten healthy adult female albino rats of Wistar strain (procured from Animal Facility Center of Pharmacology department of Faculty of Pharmacy and Alternative Medicine, The Islamia University of Bahawalpur, Bahawalpur, Pakistan) were used to conduct study. They were divided into two groups, i.e. group A and group B, with each group having five animals. Animals transient room (room temperature: 25 ± 2 °C, relative humidity: 65 ± 5%, 12 h light/dark cycle) was used for housing animals. All animals were provided with water *ad libitum* and balanced diet. The experimental protocol used in the present research was reviewed and approved by Pharmacy Research Ethics Committee of The Islamia University of Bahawalpur, Pakistan (23–2016/PREC). Female albino rats were selected as they are generally more sensitive than males. They were chosen randomly, marked for identifica.tion, and kept in cages for at least 5 d in an animal transient room. All the animals were nulliparous, non-pregnant, 9–10 weeks old with weight ranged approximately 210 ± 10 g. Guidelines established in guide for Care and Use of Laboratory Animals and animal welfare act was strictly followed (Nevalainen, 2007).

##### Preparation of doses

Hydrogel formulation FMA5 having maximum DLE and ultimately cumulative drug release was selected to assess its safety profile in healthy adult rats as compared to normal saline administered as control. Toxicity study was conducted with the doses that are recommended by the Organization for Economic Co-operation and Development (OECD) guidelines 420 for investigating the oral toxicity of any new substance. Experimental animals were divided into two groups (*n* *=* 5). Group A was used as control and has been administered 1 mL/100 g body weight 0.9% saline per orally. Group B were given developed FMA5 hydrogel at a dose of 5 g/kg bodyweight. Group B was given hydrogel in form of suspension using normal saline as vehicle by oral gavage, thrice at an interval of 30 min and a total volume of 1 mL/100 g body weight (Ullah et al., 2019).

##### Clinical observations

All Animals were observed individually shortly after administration of dose carefully with extra attention for the initial four hours and thereafter on daily basis for 14 d. Clinical manifestation included alteration in skin or fur, mucus membranes, eyes, salivation, tremors, diarrhea, sleep, and coma for 14-d on regular basis. Attention was given to observe responses of animals to handling, stereotypic activities like excessive grooming, repetitive circling, alteration of gait and posture. Calculation of feed intake was done as g/animal/day whereas calculation of water intake was done as mL/animal/day (Akhlaq et al., 2020).

##### Biochemical blood analysis

On day 15, which is the end of experimental period for acute oral toxicity study of developed hydrogels, animals in both groups were anesthetized with ketamine (22 mg/kg body weight) and then sacrificed by cervical decapitation. Blood samples for hematological examination were collected through cardiac puncture from posterior vena cava in Ethylene Diamine Tetra acetic acid (EDTA) blood collection tube (Khan et al., 2020).

##### Histopathological study

For conducting histopathological investigation, animals in both groups were sacrificed at the end of experimental study. After this, vital organs, i.e. heart, liver, kidney, spleen, and lungs were removed, flushed with ice-cold saline and weighed. Gross inspection of these isolated organs was conducted for any abnormality and presence of lesions. Later they were transferred into 10% formaldehyde solution for 48 h. The tissues were then fixed in molten paraffin wax, segmented into 5 µm, and stained by hematoxylin and eosin. After histological staining, observation of slides was done under optical microscope (Farid-ul-Haq et al., 2020).

##### Statistical analysis

Results are indicated as mean ± standard error of mean (SEM). IBM SPSS Statistics version 20 program (SPSS Inc., Armonk, NY) was used for statistical analysis of acute oral toxicity study results. The difference between two groups was determined by one-way analysis of variance (ANOVA) with Tukey test. A value of *p* < .05 was regarded statistically significant.

### Pharmacokinetic study in rabbits

#### High-performance liquid chromatographic analysis

A rapid, sensitive and simple reversed-phase high-performance liquid chromatographic (HPLC) method has been developed and validated for the determination of ACV in rabbit plasma. Similar to acute oral toxicity study, hydrogel formulation FMA5 having maximum dDLE and ultimately cumulative drug release was selected for pharmacokinetic evaluation. BDS C18 column was used to conduct analysis using ammonium dihydrogen phosphate buffer (50 mM) and methanol as mobile phase (98:2), with pH adjusted to 2.5 using orthophosphoric acid. Flow rate was kept at 1 mL/min. Selective precipitation of plasma proteins was done by the addition of 5% perchloric acid. Direct injection of supernatant was given into a BDS C18 column and ACV was detected at 256 nm. The limit of detection for ACV in plasma was estimated as 15 ng/mL whereas the limit of quantitation was calculated as 25 ng/mL (Malik et al., 2019).

#### Animals and drug administration

Pharmacokinetic studies were conducted on 24 healthy adult albino rabbits (2.0–2.5 kg) which were kept at 12/12 h light/dark cycle with free access to food and water. Animals transient room (room temperature: 25 ± 2 °C, relative humidity: 65 ± 5%, 12 h light/dark cycle) was used for housing animals. Two groups of rabbits, i.e. group A and group B (twelve rabbits in each group) were fasted for 12 h with free access to water. Group A was used as control and has been administered ACV in suspension form by adding ACV powder to normal saline. Group B was used as hydrogel group and has been given developed FMA5 hydrogel formulation. Both control and experimental groups were given a total dose of 20 mg/kg bodyweight of their respective formulation. Microsoft^®^ Office Excel 2019 program was used for estimation of ACV concentration in rabbit plasma in hydrogel as well as control groups. Non-compartmental pharmacokinetic model was used to analyze pharmacokinetic parameters, such as area under the concentration-time curve (AUC_total_), time to reach maximum drug concentration (T_max_), peak plasma drug concentration (C_max_), half-life (*t*_1/2_), and clearance (Cl). Calculation of pharmacokinetic parameters was done using scientific application package Kinetica^®^ version 4.1.1 (Thermo Electron Corporation, Waltham, MA). Pharmacokinetic data were statistically analyzed by one-way ANOVA. Among calculated parameters, *p* value < .05 was regarded statistically significant (Khan and Anwar, 2021).

## Results and discussion

### FTIR spectroscopy

**Figure 1.**
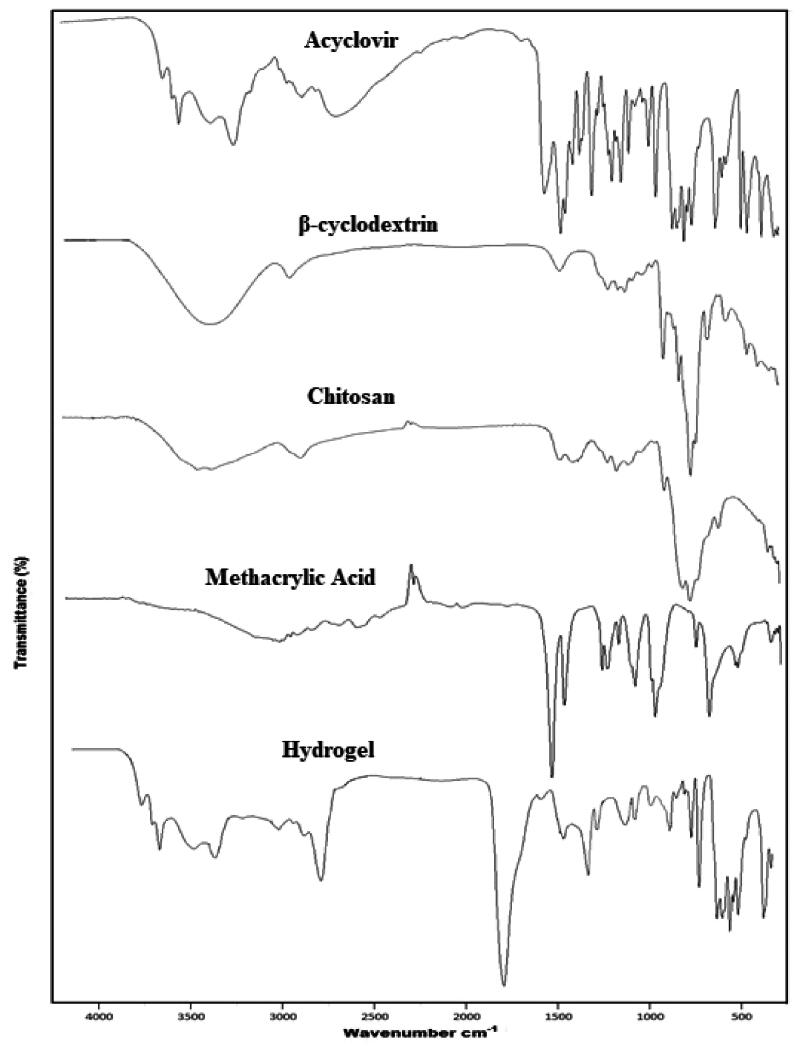
FTIR curve of acyclovir, β-cyclodextrin, chitosan, methacrylic acid, and β-CD/CS-co-poly(MAA) hydrogel.

The formation of β-CD/CS-co-poly(MAA) hydrogel was confirmed by FTIR spectra as shown in Figure 1. The FTIR spectra of ACV showed peak at 344 cm^−1^ and 3178 cm^−1^ due to N–H and O–H stretching vibrations. The aliphatic C–H stretching vibrations, C=O stretching vibrations and N–H bending were noted at 2687 cm^−1^, 1707 cm^−1^, and 1630 cm^−1^, respectively, for ACV. Pure polymer CS showed absorption band at 3358 cm^−1^ indicating overlapping of –OH and symmetric N–H stretching vibrations. The bands observed at 1644 cm^−1^, 1605 cm^−1^, and 1375 cm^−1^ are due to carbonyl stretching vibration (amide-I), N–H stretching vibration (amide-II) and the C–N stretching vibration (amide-III) of pure polymer.

In FTIR spectra, β-CD represented broad transmittance peak at 3303 cm^−1^ due to –OH stretching vibrations, asymmetric stretching vibrations of –CH were represented at 2928 cm^−1^ whereas asymmetric stretching vibrations of C–O were represented at 1633 cm^−1^. A band at 1021 cm^−1^ was due to coupling vibrations of C–O and C–C. Monomer MAA represented characteristic band at 2987 cm^−1^ that showed the presence of methyl C–H asymmetric stretching. Another important band in the range of 1698 cm^−1^ has been designated for carboxylic acid, whereas band at 1636 cm^−1^ represented C=C stretching vibrations.

The FTIR spectrum of developed β-CD/CS-co-poly(MAA) hydrogels exhibited a higher and broader peak at about 1770 cm^−1^. This peak has been assigned to C=O stretching vibration of the ester group on polymeric backbone and resulted from esterification between hydroxyl group on CS and carboxyl group on MAA. A band at 1573 cm^−1^ and 1410 cm^−1^ have been assigned to asymmetric and symmetric stretching of C=O. Moreover, the characteristic absorption bands of amide-II and amide-III (1605 and 1375 cm^−1^) of CS could not be found. Such information confirms that –NH_2_ from CS took part in the grafting reaction. A characteristic band observed at 897 cm^−1^ was due to the vibration of α-(1 → 4) glucopyranose ring of β-CD, indicating grafting of β-CD on polymeric backbone. Furthermore, characteristic bands were observed in developed polymeric network with slight shifting at 3440, 3150, and 2695 cm^−1^, representing N–H and O–H, and C–H stretching vibrations, respectively. These vibrations represented presence of ACV in polymeric network of β-CD, CS, and MAA. Thus, in FTIR spectra of developed hydrogels, most of the characteristic peaks of components persisted with insignificant deviation (Aflaki Jalali et al., 2016). Moreover, slight shifting of some of the characteristic peaks of pure components and appearance/disappearance of some of peaks in polymeric network is an indication of formation of new polymeric network (Maia et al., 2012). It also confirms that developed polymeric network is chemically compatible with the model drug, ACV (Cirri et al., 2012).

### Thermal analysis

**Figure 2.**
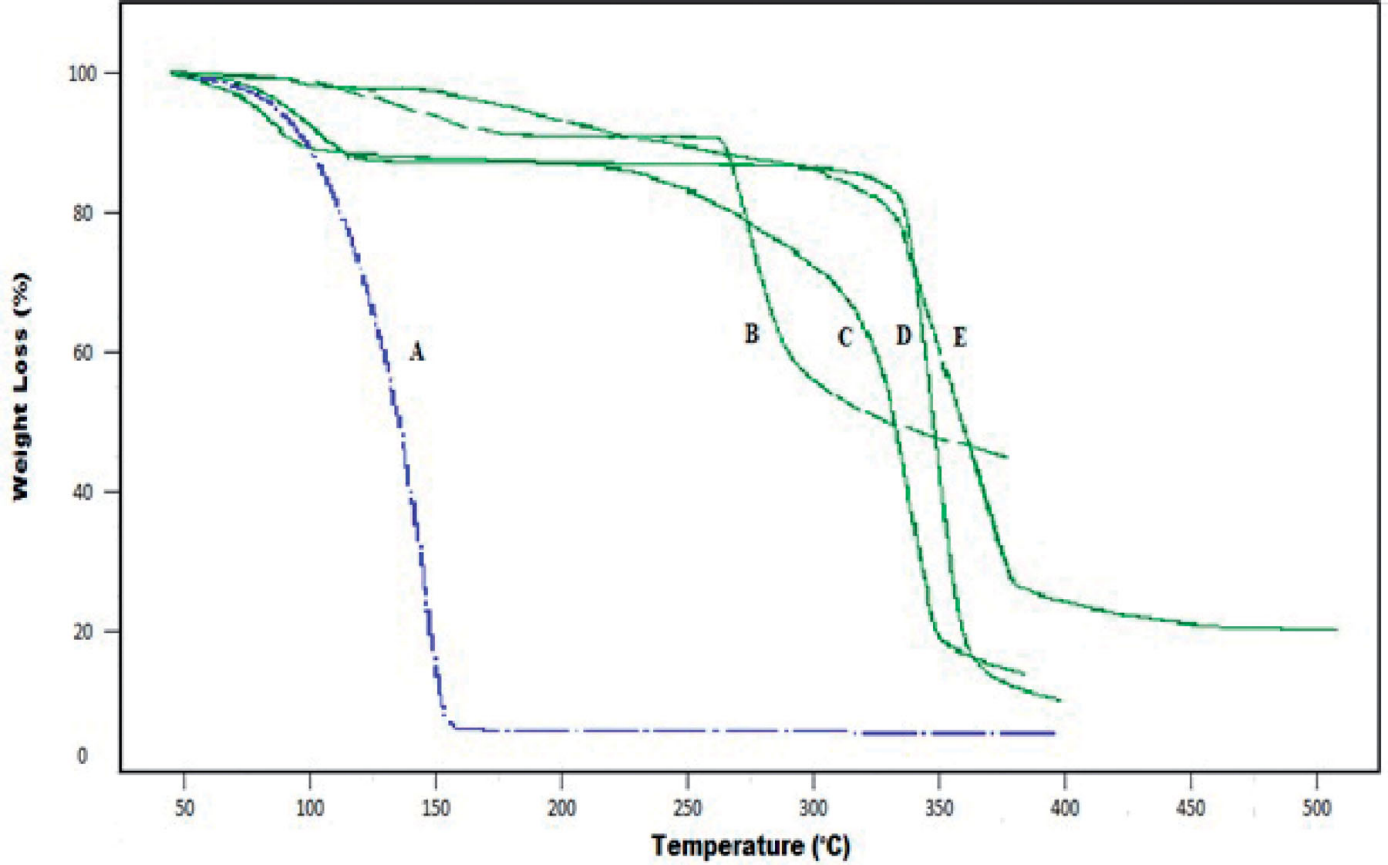
TGA curve of (A) methacrylic acid (B) acyclovir (C) β-cyclodextrin (D) chitosan (E) β-CD/CS-co-poly(MAA) hydrogel.

Figure 2 shows TGA thermogram of developed hydrogel and individual reactants representing weight loss at different temperature range. In case of TGA thermogram of pure ACV, initial decay in thermogram starts at 99 °C while further degradation happened at higher temperatures, i.e. at 306 °C. The TGA thermogram of pure polymer CS represents weight loss at two stages, i.e. initially due to loss of bound water at about temperature 91 °C and later 35%, representing decomposition of its major structure at temperature range of 317 °C. At temperature range of 355 °C CS also shows a residue mass of about 17%. Pure polymer β-CD decomposes in two steps. Despite the fact that water evaporation is connected to initial 13% weight loss, the second prominent weight loss, i.e. 18% was observed at 327 °C. The main decomposition, i.e. 84% occurs at 359 °C. It is due to degradation of the residual polymer. TGA thermogram of MAA exhibited prominent degradation at 160 °C.

Developed hydrogels of β-CD/CS-co-poly(MAA) showed its thermal degradation in two stages. The first stage started at 340 °C with a small weight loss of 20%, corresponds to the elimination of water molecules trapped inside polymeric network. The second major 60% weight loss took place in the temperature range of 350–380 °C. After this, no further weight loss of the sample was observed up to 500 °C. Infact, the major degradation at second stage involves series of process including dehydration and depolymerization. Moreover, it also indicated initiation of decomposition process of developed hydrogel. From the TG curves, it is obvious that degradation for developed hydrogels started at elevated temperature with slower weight loss rate as compared to the individual reactants (Wei et al., 2015). Thus, it can be concluded that the thermal stability of pure components as well as model drug ACV has been improved as a result of grafting and developed polymeric network is more stable than components (Hu et al., 2014). Higher thermal stability represented strong inter-molecular interaction existed between components as a result of cross-linking (Ray et al., 2010).

**Figure 3.**
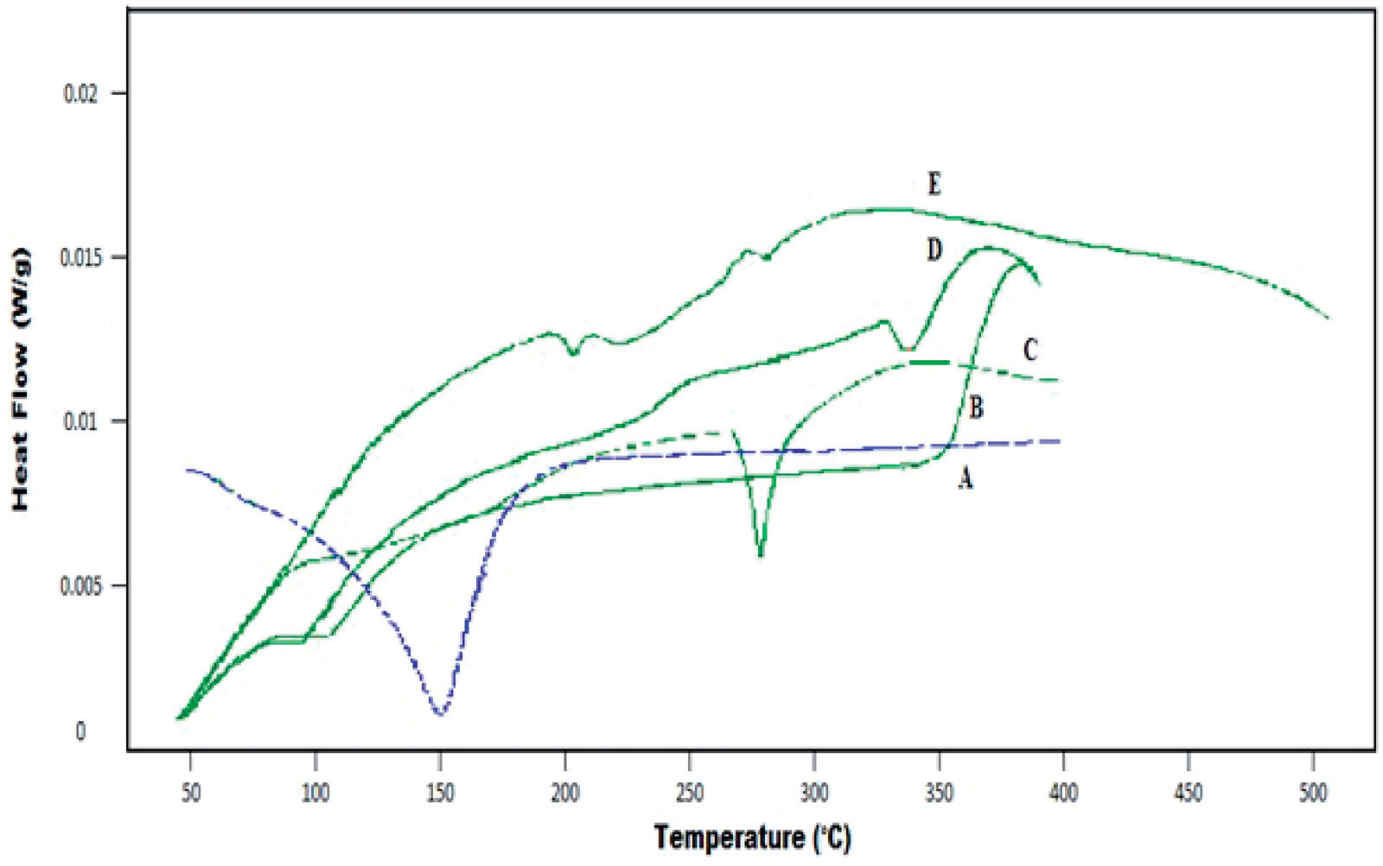
DSC curve of (A) methacrylic acid (B) β-cyclodextrin (C) acyclovir (D) chitosan (E) β-CD/CS-co-poly(MAA) hydrogel.

Figure 3 shows the DSC thermogram of developed hydrogel and individual reactants. In case of ACV, it showed a small band at 91 °C and a sharp endothermic band at 270 °C, representing an initial moisture loss followed by its melting point temperature. The DSC curve of pure polymer CS exhibited decomposition in the temperature range of 229–341 °C with endothermic peaks, representing degradation of its amino and N-acetyl residue. β-CD revealed two endothermic peaks, first at 107 °C and second at 329 °C.

Developed β-CD/CS-co-poly(MAA) hydrogels, two small endothermic peaks were observed at elevated temperature, i.e. 203 °C and 278 °C, respectively. These small endothermic peaks in DSC thermogram represent glass transition temperature (Tg) of developed polymeric network. The higher glass transition temperature (Tg) of developed polymeric network as compared to individual components indicated enhanced crosslinking density between reaction components which reduces the flexibility and ability of polymeric chain to undergo segmental motion (Ebrahimi et al., 2015). Moreover, this high-crosslinking density has resulted due to either covalent bonding or higher intermolecular hydrogen bonding, leading to higher glass transition temperature and thermal stability of developed polymeric network (Nair et al., 2014; Mahmood et al., 2018).

### Scanning electron microscopy (SEM)

**Figure 4.**
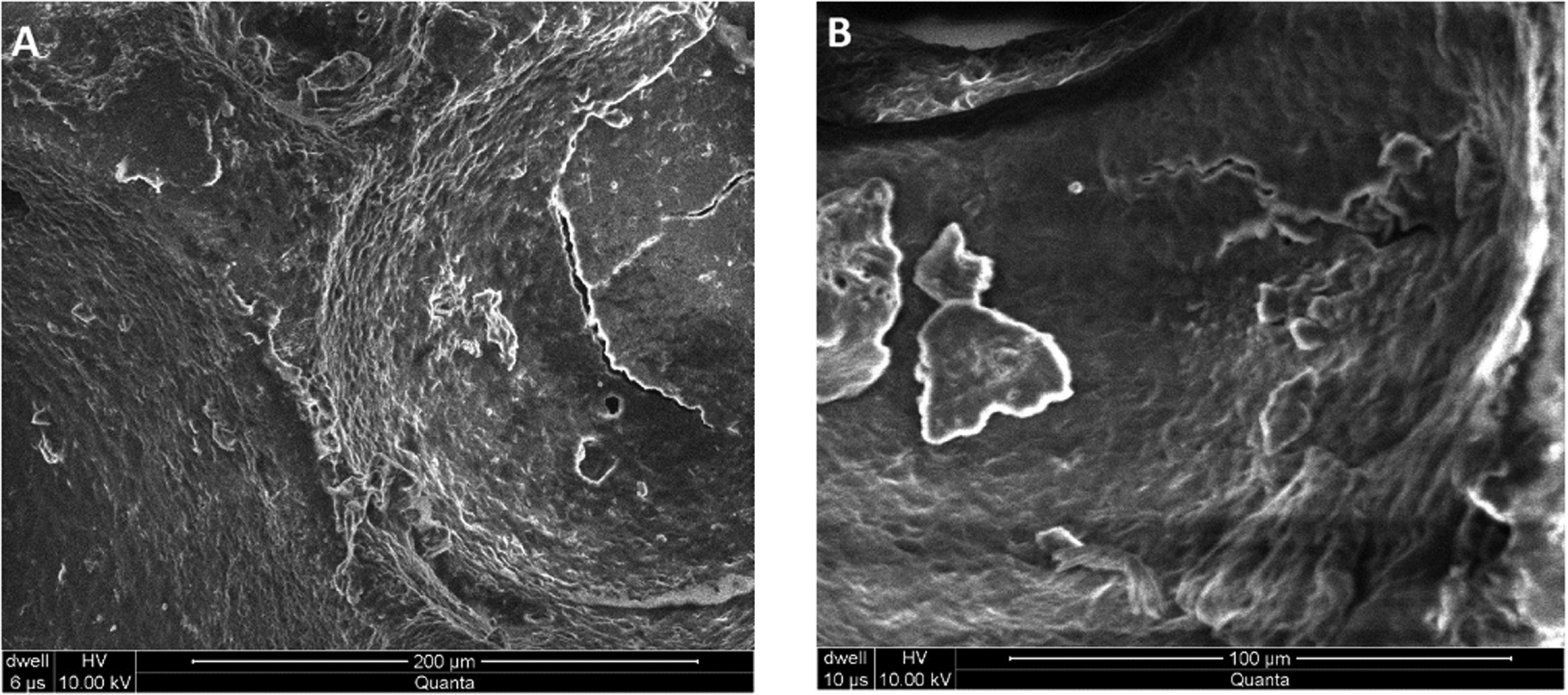
SEM images of ACV loaded β-CD/CS-co-poly(MAA) hydrogel disk (A) at magnification of 200 µm (B) at magnification of 100 µm.

The surface morphology of drug-loaded hydrogels was analyzed by scanning electron microscopy (SEM). As shown in Figure 4, the SEM photograph of these hydrogels exhibited an irregular, slightly porous surface containing characteristic large wrinkles and cracks. These cracks and wrinkles on the hydrogel surface might be caused by partially collapsing of the polymeric gel network during drying (Sinha et al., 2015). Moreover, polymeric debris was seen on the hydrogel surface, which could be due to simultaneous formation of hydrogels and synthesis of polymeric-blend matrix. The presence of drug crystals on the hydrogel surface might be due to their migration along with water to the surface during drying. The reason of low porosity can be attributed due to the presence of high-crosslinking density and drying method used in our study (Nayak et al., 2014). Hot oven-dried hydrogels exhibit less porous structure when compared with vacuum-freeze-dried hydrogels (Shafaghi et al., 2014).

### Powder X-ray diffraction (PXRD) analysis

**Figure 5.**
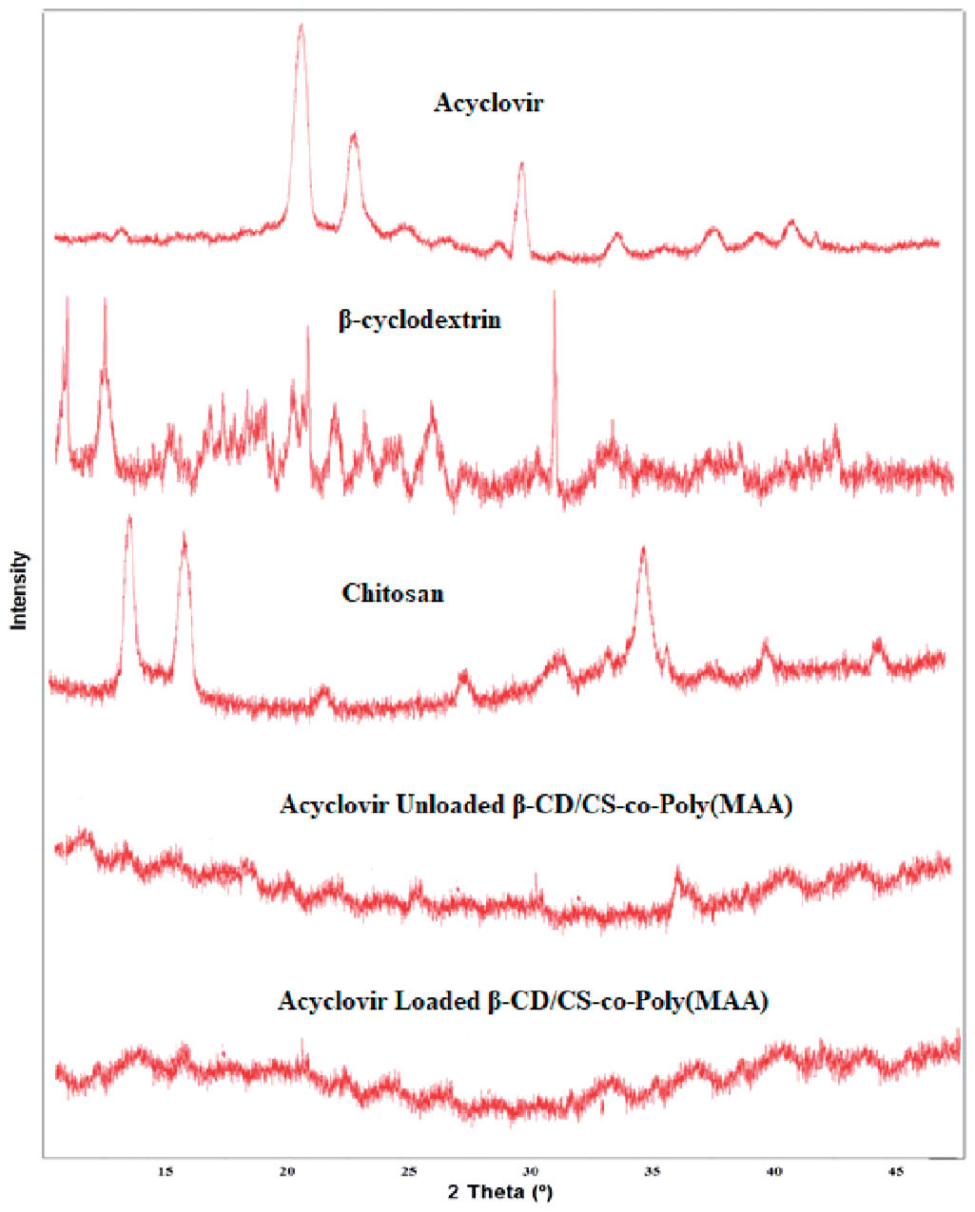
PXRD pattern of acyclovir, β-cyclodextrin, chitosan, acyclovir unloaded hydrogel, acyclovir loaded hydrogel.

To investigate physical state of drug in the developed hydrogels, PXRD analysis has been performed. PXRD diffractogram of model drug ACV, pure polymer, and hydrogels have been shown in Figure 5. ACV exhibited sharp, intense and characteristics peaks at 2*θ* = 18.50°, 21.50°, and 30.50° whereas PXRD analysis of pure polymer CS revealed its crystalline structure representing a strong diffraction peaks at around 13.06° and at 17.56°, respectively. Moreover, crystalline state of β-CD was also evident in its PXRD diffractogram, displaying intense and characteristics peaks at 2*θ* = 5.75°, 7.90°, 22.23°, 23.03°, 24.85°, and 28°.

Compared with CS and β-CD, the sharp and characteristic peaks disappeared in PXRD analysis of drug unloaded hydrogel disk. Instead they were replaced with attenuated, low intensity, and broad peaks, indicating that after grafting, the original crystalline structures of polymers have been altered. Moreover, these observations also indicated occurrence of the grafting process in a haphazard manner along polymeric backbone and consequently destroying the intermolecular hydrogen bonds (Hu et al., 2014). Further, PXRD analysis of ACV loaded hydrogel disk also showed presence of small dispersed peaks instead of sharp peaks. Thus, it can be concluded that disappearance of ACV characteristic peaks in PXRD analysis hinted its inability to form drug crystals and, therefore, molecular dispersion of drug within the developed polymeric network during preparation process (Bajpai et al., 2016).

### Swelling studies

**Figure 6.**
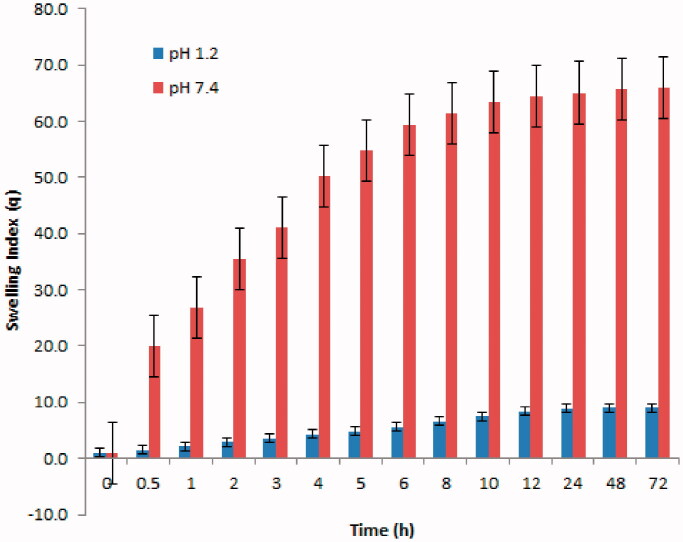
Mean swelling index of β-CD/CS-co-poly(MAA)hydrogels at pH 1.2 and pH 7.4.

Results of the mean swelling studies are depicted in Figure 6. The results clearly revealed very small volume change of hydrogel in SGF, while drastic volume changes of hydrogels in SIF. The significant swelling ratio at higher pH is very important for efficient and desirable drug release profile. It can be assumed that swelling behavior of developed hydrogels depends upon the presence of functional groups that could be ionized or protonated, hydrophilic–hydrophobic interactions, and relaxation of polymeric chain.

At pH 1.2, the swelling ratio of hydrogel depends on protonation of NH_2_ group of CS. However, due to crosslinking of CS with polymeric network, number of NH_2_ groups in polymeric network has been decreased significantly. Also at very acidic conditions (pH 1.2), a screening effect of the counter ions, i.e. Cl−, shields the charge of ammonium cations, thus prevents an efficient repulsion between them. Moreover, the carboxylic group of MAA in polymeric network remains unionized at acidic pH. Hydrogen bond formation occurred between carboxylic groups, which acted as a barrier that strengthened the polymeric network and hindered the entrance of water molecules inside the hydrogel network. All these factors have imparted physical strength to the hydrogel (Akhtar et al., 2015). Thus, hydrogel remains collapsed and therefore swelling ratio was insignificant at pH 1.2 as obvious in Figure 6.

Developed hydrogel undergoes drastic change in swelling pattern at pH 7.4. This significant swelling ratio in SIF was a result of ionization or dissociation of carboxylic group of MAA in polymeric network. As the pH of surrounding media increases above pKa of carboxylic acid of MAA, they ionize and attract cations from media to replace the H + ions. Thus an increase in the degree of ionization of carboxylic group of MAA at higher pH contributes to an increase in charge density on polymeric network, greater electrostatic repulsion among negatively charged (COO^−^) groups and greater expansion of the network (Bashir et al., 2016). This all leads toward relaxation of macromolecules and increased swelling dynamics of developed hydrogels. Additionally, the carboxylate anions induce more hydrophilicity in the polymeric network thus contributing to enhanced swelling ratio of hydrogels (You et al., 2015).

### Effect of different components of hydrogel on swelling

**Figure 7.**
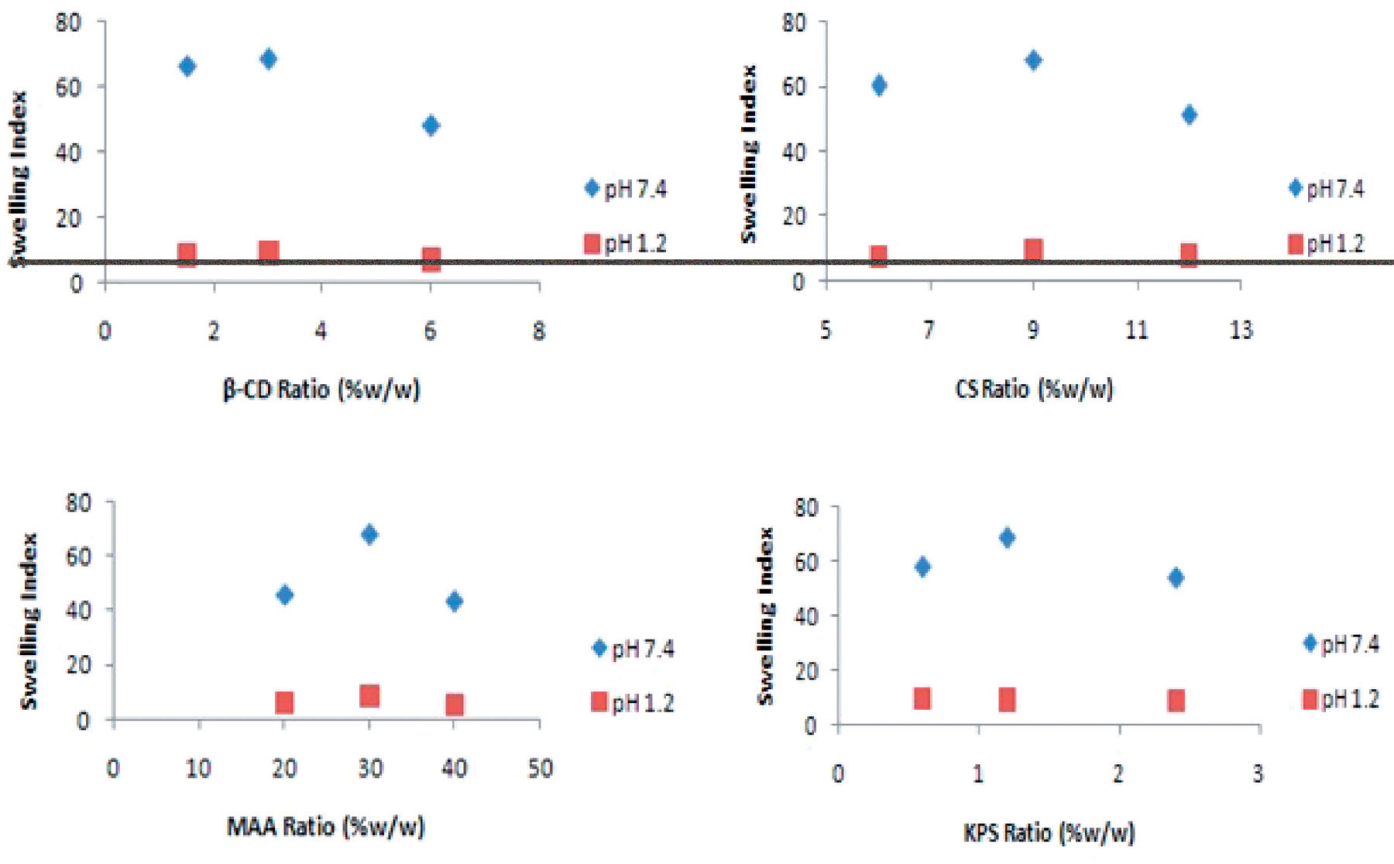
Effect of different concentration of β-cyclodextrin, chitosan, methacrylic acid, and potassium persulfate on swelling index of β-CD/CS-co-poly(MAA) hydrogels.

Figure 7 shows that the concentration of reactants used in the reaction mixture plays a significant role in governing the swelling behavior of synthesized hydrogels.

Synthesized hydrogels were investigated by varying the concentration of pure polymer CS from 6% to 12%. It is obvious from the figure that the swelling dynamics increased with increase in CS concentration from 6% up to 9%. When the CS content exceeded 9%, the swelling ratio gradually decreased. All developed hydrogels were expected to show increased swelling ratios due to hydrophilic nature of CS, which would increase the water absorption of the hydrogel. However, our results showed that the hydrogel with 9% concentration of CS achieved the highest swelling ratio. The swelling ratios then decreased as CS concentrations increased from 9% up to 12%, which indicated a less swollen hydrogel. The decrease in swelling ratio on increasing CS ratio beyond 9% might be due to the fact that as concentration of CS increases, more space within the hydrogel network was filled up by CS. This space fill-up resulted in the formation of a more rigid and compact hydrogel structure, which provides resistance for buffer solution to penetrate. Hence, with decrease in penetration of buffer solution inside polymeric network, swelling ratio decreases.

Increasing concentration of β-CD in developed hydrogel formulations from 1.5% to 3% have led to an increase in the swelling index of hydrogels. This increase of swelling index may be attributed to the hydrophilic nature of β-CD and the increased number of functional units available for grafting, thus leading to enhanced grafting efficiency. However, on further increase in concentration of β-CD, from 3% to 6% the steric effect of β-CD outweighs the ionic effect of ionic groups of the polymer, leading to a lower swelling ratio (Ooi et al., 2016).

Swelling ratio increased with increase in monomer (MAA) concentration from 20% up to 30%. When the MAA content exceeded 30%, the swelling ratio gradually decreased. Hydrogel formulations with MAA concentration 30% exhibited higher swelling ratio in comparison to formulations with less concentration of MAA, i.e. 20%. The carboxylic groups of MAA were mainly responsible for swelling tendency in hydrogels. It has been observed that with increase of MAA concentration, the number of carboxylic group also increased within the hydrogel structure. These hydrophilic moieties present on the monomer favored water absorption (Wang et al., 2009). However, further increase in concentration of MAA from 30% to 40% has resulted in reduction in swelling ratio. This fact is attributed due to formation of a more rigid polymeric network by inter–intra polymer chain reactions. As a consequence, flexibility and number of hydrophilic groups exposed to the medium decreases. Moreover, incorporation of very high concentration of monomer promotes their self-crosslinking, hence, prevents accessibility of more solvent in the developed matrix (Jalil et al., 2017).

Synthesized hydrogels were also investigated with varying amounts of initiator KPS in the range 0.8–2.4 g, respectively. Swelling dynamics increased with increase in initiator concentration up to 1.6 g and then it begins to decrease with further increase in KPS content. Initially, when the concentration of initiator is 0.6 g, the minimum swelling dynamics of resulting hydrogel may be due to the fact that the network does not form efficiently with a small number of free radicals. However, with increase in KPS content, the number of primary free radicals increases and the polymeric chains with relatively higher molecular weight are produced. These macromolecular chains, when brought in contact with buffer media, extended or relaxed to a greater extent, thus occupying larger space and letting more and more solvent in. This results in increase in swelling dynamics. However, when the KPS concentration is increased up to 2.4 g, there is considerable decrease in swelling behavior of hydrogels. This may be attributed to the fact that the concentration of free radicals becomes so high that the growing macromolecular chains are terminated at a faster rate. This results in the formation of low molecular weight polymeric segments within the hydrogel network or shorter graft chains that could not undergo extension and relaxation to allow surrounding media to penetrate. Thus, the resulting hydrogels demonstrated a decrease in swelling dynamics (Ostrowska-Czubenko et al., 2015).

### Drug-loading efficiency (%DEE) and drug release studies

%DEE and drug release behavior of developed hydrogels at both pH 1.2 and 7.4, respectively, have been shown in Table 2. Drug loading efficiency (%DEE) has been found to be oscillating within 75.56–89.65%. Moreover, the data tabulated in Table 2, also indicates that the hydrogel formulation which have shown maximum swelling ratio exhibited the highest loading efficiency for ACV and ultimately percent release of drug, i.e. FMA5. This trend is consistent with all hydrogels (Jana et al., 2016).

**Table 2.** Drug entrapment efficiency (%DEE) and percentage drug release at pH 1.2 and pH 7.4 for a period of 24 h.

Formulation code	Drug entrapment efficiency (%DEE)	Percentage release of acyclovir	
		(For 24 h period)	
		pH 1.2	pH 7.4
FMA1	75.56	10.87	67.79
FMA2	82.7	16.98	80.22
FMA3	77.93	11.87	73.44
FMA4	78.25	12.96	75.7
FMA5	89.65	19.91	87.57
FMA6	79.74	13.47	76.55
FMA7	87.58	18.36	85.02
FMA8	84.64	16.51	83.33
FMA9	80.95	15.81	78.81

As FMA5 hydrogel has the highest loading efficiency of 89.65%, the possible explanation of maximum drug release is enhanced availability of ACV, leading to its improved percent release. Moreover, in swollen state, the drug residing in free space of the polymeric network behaves as a plasticizer, leading toward greater flexibility of hydrogels. Hence, solvent or buffer molecules could enter or leave the hydrogel as more spacious path for diffusion and movement of media across polymeric network is available, thereby facilitating the release of incorporated ACV across polymeric network (Guru et al., 2013).

**Figure 8.**
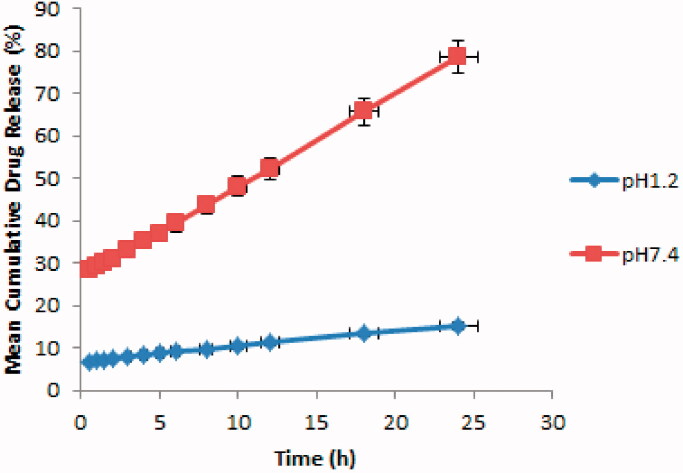
Mean cumulative drug release of β-CD/CS-co-poly(MAA) hydrogels at pH 1.2 and pH 7.4.

Figure 8 shows mean cumulative drug release, which was found to be much higher in SIF than in SGF. Thus it can be said that the drug release rate from developed hydrogels gives a reflection of their swelling behavior (El-Sherbiny, 2010).

The results of curve fitting into various important kinetic models are given in Table 3. When respective correlation coefficients of these polymeric networks in SGF and SIF were compared, the ACV release from these hydrogels was found to follow zero-order kinetics (Anraku et al., 2015). The value of release exponent (*n*) determined from *in vitro* drug-release data of developed hydrogels containing ACV ranged from Fickian mechanism in SGF to non-Fickian SIF, respectively.

**Table 3.** Determination of regression coefficient *R*^2^, K, and release exponent ‘n’.

Sample code		Zero-order kinetics		First-order kinetics		Higuchi model		Korsmeyer–Peppas model		
		*R* ^2^	*K*	*R* ^2^	*K*	*R* ^2^	*K*	*R* ^2^	*K*	*n*
FMA1	1.2	0.99	0.165	0.266	0.146	0.961	3.645	0.932	4.786	0.308
	7.4	0.995	1.519	0.26	0.262	0.943	17.923	0.964	17.576	0.514
FMA2	1.2	0.991	0.512	0.37	0.165	0.979	4.652	0.983	5.239	0.419
	7.4	0.997	2.295	0.293	0.267	0.962	19.264	0.976	19.798	0.482
FMA3	1.2	0.99	0.252	0.253	0.154	0.967	3.995	0.95	5.047	0.334
	7.4	0.999	1.998	0.278	0.264	0.951	18.274	0.971	17.397	0.534
FMA4	1.2	0.99	0.283	0.323	0.153	0.959	3.98	0.966	5.037	0.337
	7.4	0.997	1.886	0.281	0.264	0.931	18.259	0.961	16.676	0.564
FMA5	1.2	0.991	0.735	0.45	0.167	0.968	4.713	0.977	4.117	0.593
	7.4	0.998	2.603	0.315	0.268	0.944	19.295	0.966	15.882	0.636
FMA6	1.2	0.991	0.294	0.334	0.152	0.963	3.955	0.952	4.927	0.349
	7.4	0.998	1.836	0.292	0.263	0.943	18.255	0.977	18.452	0.493
FMA7	1.2	0.993	0.612	0.424	0.163	0.959	4.473	0.947	4.23	0.539
	7.4	0.998	2.243	0.319	0.265	0.936	18.693	0.972	16.223	0.598
FMA8	1.2	0.99	0.494	0.376	0.163	0.971	4.456	0.956	4.423	0.505
	7.4	0.997	2.232	0.305	0.266	0.93	18.822	0.959	16.995	0.571
FMA9	1.2	0.992	0.443	0.37	0.161	0.961	4.35	0.952	5.176	0.382
	7.4	0.998	2.006	0.306	0.262	0.934	18	0.966	17.352	0.525

### Acute oral toxicity study

#### Clinical observations

Table 4 demonstrates the impact of oral administration of hydrogels on body weight, food and water utilization, behavior pattern, and toxicity associated symptoms in both experimental and control group. No formulation-related adverse effects were observed on health status of the rats in experimental group. None of the animal displayed any indication or sign of morbidity (Ubaid and Murtaza, 2018). There was absence of mortality during the whole observational period and no significance variation in the body weights, feed and water consumption was observed in hydrogels exposed group in comparison to control group. This proposes that hydrogel intake causes no obvious adverse effect or impairment on the health condition of experimental animals (Ji et al., 2010; Zahra et al., 2011; Sharma et al., 2012). Rats have shown sensitivity and normal behavior to touch, sound, light, and other external stimuli. Corneal reflex, gripping strength, righting reflex were present. Animal feces were in regular form without any change in color and devoid of any pus cell (Wang et al., 2010). Based on above observations, it has been clearly indicated that no harmful effects have been observed on physical characteristics, growth and general behavior of the rats after oral administration of developed hydrogels (Pour et al., 2011; Erum et al., 2015). As indicated by globally harmonized system (GHS), if chemical that is to be tested has LD50 value greater than 2000 mg/kg dose, than it is classified under ‘Category 5’ with toxicity score ‘zero.’ As no morbidity or mortality has been observed in hydrogel treated group B similar to control group A, so prepared hydrogels with a dose of 5 g/kg body weight can be categorized under Category 5, representing zero grade toxicity with higher safety margin and biocompatibility (Bulgheroni et al., 2009).

**Table 4.** Clinical observation of control and hydrogels treated rats for acute oral toxicity study.

Observation	Group A	Group B
	Mean ± SEM	Mean ± SEM
Body weight (g)		
Pre-treatment	212 ± 0.86	214 ± 1.46
Day 1	212 ± 1.02	214 ± 0.40
Day 7	223 ± 0.93	222 ± 2.18
Day 14	237 ± 4.13	236 ± 1.90
Water intake		
(mL/animal/day)		
Pre-treatment	36 ± 0.60	37 ± 0.51
Day 1	38 ± 0.45	39 ± 0.45
Day 7	39 ± 1.96	40 ± 1.30
Day 14	41 ± 2.11	39 ± 1.64
Food intake (g/animal/day)		
Pre-treatment	15 ± 0.55	16 ± 0.86
Day 1	16 ± 0.54	15 ± 0.70
Day 7	15 ± 0.25	16 ± 0.60
Day 14	19 ± 0.49	17 ± 0.68
Signs of illness	–	–
Dermal toxicity	–	–
Dermal irritation		
Ocular toxicity	–	–
Eye Irritation		
Lacrimation	–	–
Salivation	–	–
Convulsions	–	–
Hyperactivity	–	–
Touch response	+	+
Corneal reflex	+	+
Righting reflex	+	+
Gripping strength	+	+
Alertness	+	+
Mortality	–	–

Results are expressed as mean ± SEM of 5 rats in each group. Group A – Control, Group B – FMA hydrogel. Both at a dose of 5 g/kg bodyweight. − Sign indicates lack or absence of specified observation. + Sign indicates presence of specified observations. All values have *p* > .05, indicating statistically insignificant results.

#### Biochemical blood analysis

The results of different parameters calculated in biochemical blood analysis have been shown in Table 5. All hematology parameters of group B are in the standard reference range, similar to the control group A values, indicating that the developed hydrogels are likely to be nontoxic (Diallo et al., 2010). Although some parameters show significant difference between the animal groups due to inter-subject variation, none of the biochemical parameter lies outside normal reference range of experimental animals (Barkat et al., 2018). It has been observed that administration of developed hydrogels did not alter any of above biochemical parameters to toxic level. All of them were present in normal reference range (Tan et al., 2013; Uma et al., 2013; Turkmen and Dogan, 2020).

**Table 5.** Biochemical parameters of control and hydrogel treated rats for acute oral toxicity study.

Biochemical blood analysis				
Hematology parameters	Unit	Group A	Group B	*p* Value
		Mean ± SEM	Mean ± SEM	
Hemoglobin	g/dL	14.5 ± 0.10	14.2 ± 0.07	*p* > .05
Hematocrit	%	42.4 ± 0.12	42.3 ± 0.23	*p* >.05
Red blood cells	10^6^/µL	8.61 ± 0.05	8.59 ± 0.04	*p* > .05
Platelets	10^3^/µL	996 ± 1.95	994 ± 2.00	*p* > .05
White blood cells	10^3^/µL	3.6 ± 0.11	5.5 ± 0.07	*p*< .05
Monocytes	%	2.19 ± 0.02	2.09 ± 0.06	*p* < .05
Neutrophils	%	27.0 ± 0.22	26.5 ± 0.08	*p* < .05
Lymphocytes	%	76.0 ± 0.16	77.6 ± 0.06	*p* < .05
MCV	fL(µm^3^)	53.5 ± 0.13	53.6 ± 0.11	*p* > .05
MCH	pg	18.8 ± 0.03	18.9 ± 0.01	*p* > .05
MCHC	g/dL	33.5 ± 0.11	33.5 ± 0.11	*p* > .05
Serum biochemistry parameters				
Triglyceride	mg/dL	72.73 ± 0.16	72.56 ± 0.09	*p* > .05
Cholesterol	mg/dL	59.60 ± 0.07	59.67 ± 0.06	*p* > .05
Glucose	mg/dL	110.51 ± 0.02	110.61 ± 0.05	*p* > .05
Creatinine	mg/dL	0.66 ± 0.01	0.68 ± 0.008	*p* > .05
Urea	mg/dL	15.63 ± 0.14	15.68 ± 0.13	*p* > .05
Alkaline phosphatase (ALP)	U/L	119.59 ± 0.09	121.65 ± 0.09	*p* < .05
Aspartate transaminase (AST)	U/L	107.49 ± 0.14	108.74 ± 0.07	*p* < .05
Alanine aminotransferase (ALT)	U/L	30.74 ± 0.08	29.64 ± 0.12	*p* < .05
Creatinine kinase (CK)	U/L	618.66 ± 0.16	621.55 ± 0.16	*p* < .05

Results are expressed as mean ± SEM of 5 rats in each group. Group A – control, Group B – FMA hydrogel. Both at a dose of 5 g/kg bodyweight. *p* > .05 indicates statistically insignificant results whereas *p* < .05 indicates statistically significant results.

### Histopathological study

**Figure 9.**
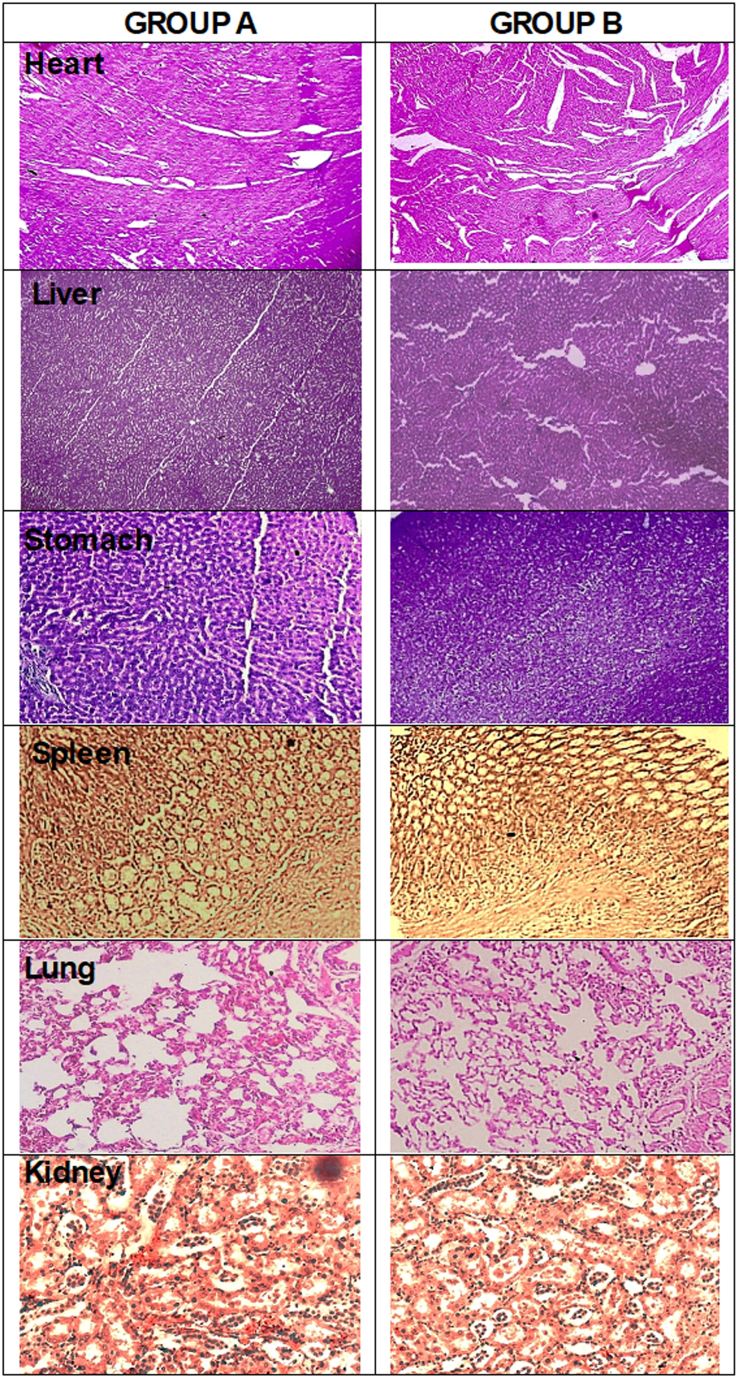
Histopathological observations of tissues from organs of group A and group B including heart, liver, spleen, stomach, lung, kidney used in acute oral toxicity study.

As indicated by Table 6, histopathological studies revealed no significant alterations in the relative weight of heart, kidneys, liver, lung, stomach, and spleen of rats treated with hydrogel in relation to the control groups. These observations indicated that administration of the hydrogels had no formulation-related toxic effect on organ weights of experimental animals (Abdullah et al., 2019). Moreover, Figure 9 indicates histopathological observation of different vital organs, i.e. heart, liver, stomach, lungs, spleen, and kidney of both groups A and B. As obvious from Figure 9, myocardial tissues of cardiac muscles were displayed orderly in both groups. They were clear and did not show any inflammatory exudate, necrosis, or hemorrhage (Khalid et al., 2021). Liver lobules of group B were present with clear dividing lines, similar to group A. No hypertrophy was obvious on hepatic sinusoid and no neutrophil, lymphocyte, or macrophage infiltration was observed, indicating lack of any toxicity (Haseeb et al., 2018). Spleen sinus was absolutely normal, without any evidence of toxicity or pathological alteration in both control and hydrogel treated group. Gastric glands of both groups were in regular arrangement and mucosa cells were clear. The tissue structure of lung of hydrogel-administered and control group are almost similar and no inflammatory cell infiltration and thickening of blood vessel walls around the bronchus was identified, representing normal physiology. The kidney tissue of group B was in normal shape similar to group A. No degeneration, bleeding and necrosis were observed within renal glomerulus. Renal, tubular or interstitial calcification was absent, representing lack of any toxicity as also observed in previous studies (Jothy et al., 2011; Barkat et al., 2020). Thus it can be assumed that all findings of histopathological observation were consistent in both group A and group B similar to hematological and biochemical biomarkers, attributed to normal functioning of vital organs. Thus, our study results indicated that dose levels up to 5 g/kg body weight of hydrogels when administered to rats produced no microscopic or macroscopic abnormality or toxic effects and was well tolerated for the 14th-day study period. Hence, developed hydrogels are nontoxic and have potential to be used as drug delivery system (Tan et al., 2013).

**Table 6.** Organ weight (g) of control and hydrogel treated rats for acute oral toxicity study.

Groups	Heart	Liver	Lung	Kidney	Stomach	Spleen
Group A	0.35 ± 0.01	3.67 ± 0.01	0.70 ± 0.01	0.83 ± 0.02	1.71 ± 0.03	0.46 ± 0.01
Group B	0.39 ± 0.01	3.68 ± 0.03	0.74 ± 0.02	0.85 ± 0.02	1.73 ± 0.03	0.45 ± 0.03

Results are expressed as mean ± SEM of 5 rats in each group. Group B-FMA hydrogel. All at a dose of 5 g/kg body weight. All values have *p* > .05, indicating statistically insignificant difference between organ weight among all groups.

### Pharmacokinetics of hydrogel formulation in rabbit

**Figure 10.**
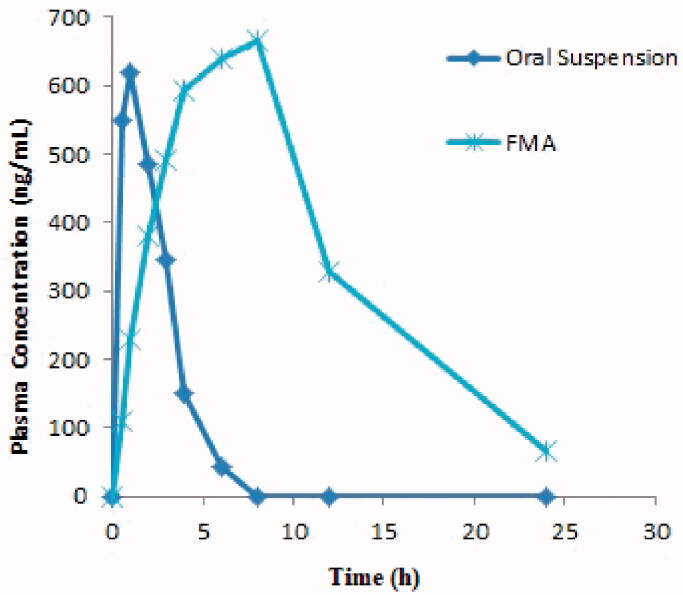
Mean plasma concentration (ng/mL) *vs.* time (h) plot of group A and group B at a dose of 20 mg/kg body weight.

Pharmacokinetic studies were conducted by administration of equal amounts (20 mg/kg of ACV) of hydrogel formulation FMA5 and ACV suspension orally in healthy rabbits. Mean plasma ACV concentration *vs.* time curve (mean ± SD) after administration of group A and group B are summarized in Figure 10, respectively. ACV was detected in plasma at 0.5 h after administration as oral suspension and 2 h following administration of hydrogel formulation FMA5 to healthy rabbits, despite the fact that concentration of ACV detected in systemic circulation was varied.

Calculation of pharmacokinetic parameters among group A and group B was done using scientific application package Kinetica^®^ version 4.1.1. Results are shown in Table 7. Rapid absorption of ACV was observed in group A (oral suspension), producing peak concentration of 630 ng/mL at 1 h. However, in group B (hydrogel group), drug absorption was extended and attained peak concentration of 675 ng/mL at 6 h. Thus, T_max_ (6 h) in group B (hydrogel group) when compared to T_max_ (1 h) of group A (oral suspension) was significantly higher (*p* < .05). This suggests that the hydrogel formulation has been able to prolong drug delivery (Al-Dhubiab et al., 2015). Further, high plasma concentrations were observed in case of hydrogel formulation, which have been kept at a relatively steady state and thereafter, a slow elimination has been conducted. Thus ACV in plasma could still be estimated in case of developed hydrogel formulation at 24 h, indicating their controlled release potential (Jana et al., 2016). However, in case ACV oral suspension, the ACV concentration in plasma was below the limit of quantitation when calculated at 8 h. The elimination half-life (*t*½) of ACV in group B was significantly higher (5 h) than group A (1 h), thus indicating of ACV release from hydrogel formulation was effective enough to maintain a therapeutic drug concentration for an extended period of time (Mahmood et al., 2016). This release trend reduces the inherent need for frequent drug administration as is the case of ACV administered as suspension to control group (Chirra et al., 2014). The AUC_total_ was found to be 5-fold higher (9858.19 ng h/mL) in case of group B, when compared with AUC_total_ of group A (1958.77 ng h/mL). This significant increase in the AUC_total_ and ultimately bioavailability of ACV loaded hydrogel formulation could be due to the release and then transport of the ACV into the systemic circulation in a sustain manner in comparison to the oral suspension (Al-Subaie et al., 2015; Wu et al., 2020). This slow and steady release avoids saturation of carrier system for transport of drug which happened in case of conventional drug delivery system of ACV, thus leading to desirable pharmacokinetic profile.

**Table 7.** Pharmacokinetics parameters of ACV after oral administration of ACV suspension to group A and hydrogel formulation (FMA5) to group B of healthy rabbits at a dose of 20 mg/kg body weight.

Sr. No	Pharmacokinetics parameters	Group A	Group B
		Oral suspension	FMA-5
		(Control)	Hydrogel
1	C_max_ (ng/mL)	630	675
2	T_max_ (h)	1	6
3	AUC_total_(ng h/mL)	1958.77	9858.19
4	*t*_1/2_ (h)	1	5
5	Clearance (L/h)	0.0102	0.00202

## Conclusion

In this study, we have evaluated the preparation of controlled drug delivery system of ACV using natural and synthetic biodegradable and biocompatible polymers through free radical polymerization technique. Results showed that the proposed method is highly useful for successful formation of hydrogels with enhanced thermal stability. Swelling dynamics, DLE, and drug release behavior were found to be dependent on pH, indicating pH-sensitive behavior. ACV release profile showed good fitting to zero-order kinetics, demonstrating controlled release pattern. Acute oral toxicity study showed no significant changes on the behavior pattern of animals, biochemical blood analysis, and histopathological study. Pharmacokinetic study revealed significant enhancement in bioavailability of polymeric network when compared with marketed oral suspension. Considering the biocompatibility, pH-dependent swelling, and drug release behavior, it can be concluded that developed hydrogel exhibited efficient drug carrier properties and could be considered as a promising platform with the aim of improving present drug delivery systems for future need.

## Animal studies

All institutional and national guidelines for the care and use of laboratory animals were followed.
