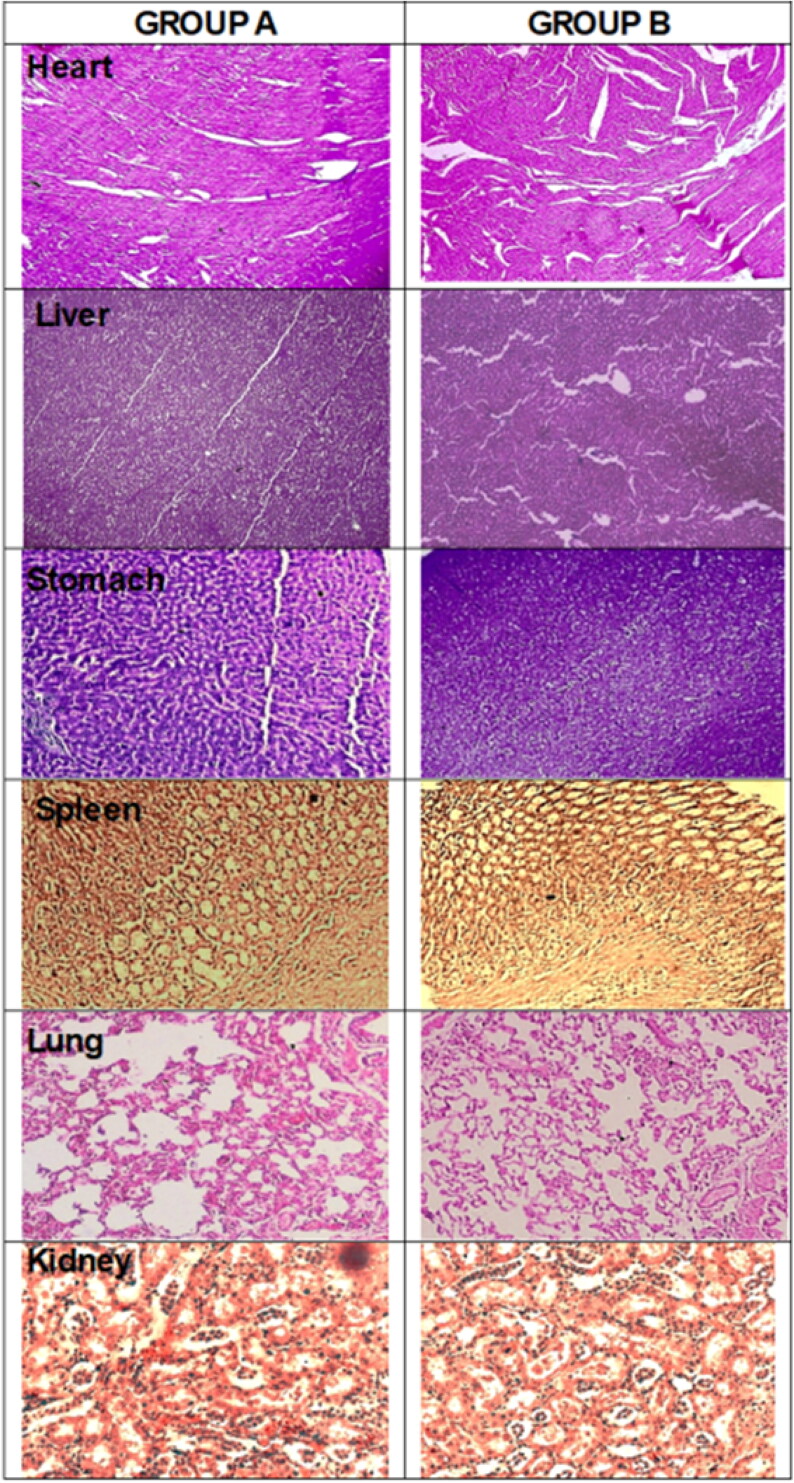# Correction

**DOI:** 10.1080/10717544.2024.2392318

**Published:** 2024-09-24

**Article title:** β-cyclodextrin chitosan-based hydrogels with tunable pH-responsive properties for controlled release of acyclovir: design, characterization, safety, and pharmacokinetic evaluation

**Authors:** Jessica Vollertsen, Mathilda Björk, Anna-Karin Norlin & Elin Ekbladh

**Journal:** *Drug Delivery*

**Bibliometrics:** Volume 28, Number 01, page 1093–1108

**DOI:** https://doi.org/10.1080/10717544.2021.1921074

When the above article published online, it contained incorrect SEM image in the Figures 4 and 9. We have corrected these errors and republished the article with the accurate images. The corrected Figures 4 and 9 are given below.

Figure 4:


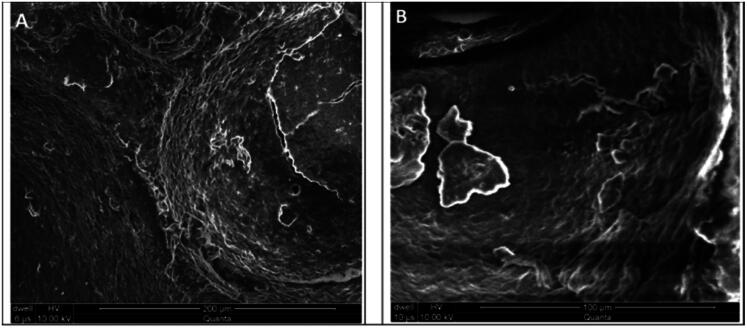


Figure 9: